# Relationship Between Intermittent Hypoxia and Type 2 Diabetes in Sleep Apnea Syndrome

**DOI:** 10.3390/ijms20194756

**Published:** 2019-09-25

**Authors:** Hiroyo Ota, Yukio Fujita, Motoo Yamauchi, Shigeo Muro, Hiroshi Kimura, Shin Takasawa

**Affiliations:** 1 Second Department of Internal Medicine, Nara Medical University, Kashihara, Nara 634-8522, Japan; ffyukio@naramed-u.ac.jp (Y.F.); motoo@naramed-u.ac.jp (M.Y.); smuro@naramed-u.ac.jp (S.M.); 2 Department of Advanced Medicine for Pulmonary Circulation and Respiratory Failure (Donation Course), Nippon Medical School Graduate School of Medicine, Bunkyo, Tokyo 113-8602, Japan; k-hiroshi@nms.ac.jp; 3 Department of Biochemistry, Nara Medical University, Kashihara, Nara 634-8521, Japan; shintksw@naramed-u.ac.jp

**Keywords:** type 2 diabetes, intermittent hypoxia, glucose-induced insulin secretion, pancreatic β cell proliferation, hepatokines, adipokines, myokines, sleep apnea syndrome

## Abstract

Sleep apnea syndrome (SAS) is a very common disease involving intermittent hypoxia (IH), recurrent symptoms of deoxygenation during sleep, strong daytime sleepiness, and significant loss of quality of life. A number of epidemiological researches have shown that SAS is an important risk factor for insulin resistance and type 2 diabetes mellitus (DM), which is associated with SAS regardless of age, gender, or body habitus. IH, hallmark of SAS, plays an important role in the pathogenesis of SAS and experimental studies with animal and cellular models indicate that IH leads to attenuation of glucose-induced insulin secretion from pancreatic β cells and to enhancement of insulin resistance in peripheral tissues and cells, such as liver (hepatocytes), adipose tissue (adipocytes), and skeletal muscles (myocytes). In this review, we focus on IH-induced dysfunction in glucose metabolism and its underlying molecular mechanisms in several cells and tissues related to glucose homeostasis.

## 1. Introduction

Sleep apnea syndrome (SAS) is characterized by repeated bouts of hypoxemia during sleep and is associated with daytime sleepiness and decline in quality of life [1]. SAS affects 32% of the adult population [2]. Type 2 diabetes mellitus (DM) is a metabolic disease characterized by reduced insulin sensitivity and increased insulin resistance, β cell dysfunction especially in glucose-induced insulin secretion, and elevated hepatic glucose production [3].

There is a significant association between SAS and type 2 DM with 15–30% of SAS patients having DM [4,5]. The relationship between SAS and type 2 DM is independent of obesity and family history of type 2 DM [6]. SAS is an independent risk factor for onset and progression of T2DM [4] and for insulin resistance [7]. Presence of type 2 DM in SAS is independent of age, sex, family history, and body habitus such as obesity [8]. The incidence of type 2 DM correlates with the severity of SAS; the correlation between hemoglobin A1c (HbA1c) values and the apnea-hypoxia index was reported to be r = 0.345, *p* = 0.016 [9]. The severity of SAS is related to the poor glycemic control in type 2 DM and a positive correlation was reported between the presence of SAS and HbA1c (r = 0.24, *p* = 0.02) [10]. SAS is characterized by recurrent upper airway collapse during sleep and, consequently, the subjection of tissues and cells to intermittent hypoxia (IH) [11]. Organs and tissues in SAS patients are exposed to varying oxygen pressures, where low oxygen pressure (hypoxemia) alternated with normoxia [12]. IH causes oxidative stress abnormalities that are similar to those seen with ischemia-reperfusion injury [13,14,15,16] and lead to redox-activated signal transduction pathways in inflammation [17,18,19]. The close relationship between nocturnal IH and impaired glucose metabolism shown in several studies suggests the possibility that IH plays a key role in the onset and progression of type 2 DM in SAS patients [20]. An association has been shown between nocturnal IH and risk of developing type 2 DM in community-dwelling Japanese participants [21]. The deleterious effects of IH on pancreatic β cell function, insulin resistance, and atherogenesis have been shown in animal studies [22,23,24]. IH, therefore, plays a key role in the pathogenesis of type 2 DM (it is described as SAS in the manuscript), and experimental studies of SAS/IH demonstrate that IH has similar effects on glucose metabolism [25]. Despite this, the pathophysiological and molecular mechanisms of IH-induced impaired glucose metabolism are incompletely understood. The present review summarizes the current understanding of the pathophysiologic and molecular mechanisms involved in the glucose metabolic dysfunction caused by IH.

## 2. Intermittent Hypoxia in Pancreatic β Cells

**Figure 1:**
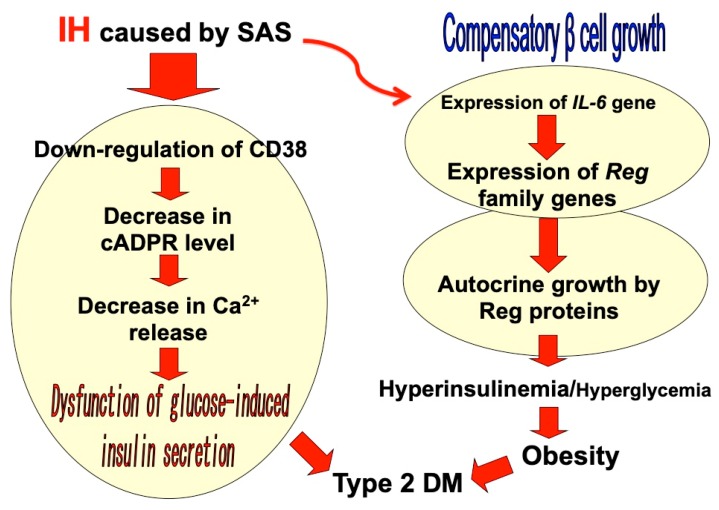
A possible model of intermittent hypoxia (IH)-induced dysfunction/proliferation of pancreatic β cells. IH in sleep apnea syndrome (SAS) patients induces β cell dysfunction by attenuation of the CD38-cADPR signal system [31,32,33,34,35,36]. IH also stimulates β cell proliferation via upregulation of *Reg* family gene expression [40]. As a result, of the malfunctioned (decreased glucose-induced insulin secretion and increased basal insulin secretion), β cell numbers are increased.

IH during sleep leads to alterations in pancreatic β cell function such as glucose-induced insulin biosynthesis, which includes preproinsulin mRNA transcription, proinsulin synthesis (translation), and insulin secretion. The progression to type 2 DM depends on the impairment of glucose-induced insulin secretion from pancreatic β cells as well as the presence of insulin resistance in peripheral target tissues and organs, including the liver, adipose tissue, and muscle. Recent reports have suggested a number of factors that may impair pancreatic β cell functioning. Hyperglycemia is known as a natural occurring and very potent factor in promoting β cell replication [26,27], which can provide an increased source of insulin to combat insulin resistance/glucose intolerance. However, IH is also reported to cause β cell replication and apoptosis without hyperglycemia [28,29], suggesting a possible mechanism by which IH acts as a β cell replication factor. On the other hand, it is reported that IH reduces β cell apoptosis through the upregulation of anti-apoptotic B cell lymphoma 2 (Bcl-2)-associated X protein (Bax) and the downregulation of apoptosis-producing Bcl-2 [30]. It is also reported that, in an animal experiment, chronic IH was a possible contributor to mitochondrial-derived reactive oxygen species in pancreatic β cell injury and dysfunction [23]. Cellular studies have demonstrated that IH significantly decreases the gene expression of cluster of differentiation (CD)38 (ADP-ribosyl cyclase/cyclic ADP-ribose [cADPR] hydrolase: EC 3.2.2.6) [31], which is an important component involved in glucose-induced insulin secretion through the mobilization of Ca^2+^ from the intracellular Ca^2+^ pool via type 2 ryanodine receptor Ca^2+^ channel, by cADPR in primary cultured rat and mouse pancreatic islets and animal model experiments [32,33,34,35,36]. IH also increased rodent pancreatic β cell replication by upregulation of the regenerating gene (*Reg*) family genes, which encode autocrine and paracrine growth factors for β cell replication [37,38,39], and by the upregulation of an antiapoptotic hepatocyte growth factor, the upregulation of which may combat the presence of β cell dysfunction and insulin resistance [40] (Figure 1).

## 3. Intermittent Hypoxia and the Liver

### 3.1. IH Induces Liver Damage

IH has been shown to cause damage of liver cells (hepatocytes) and elevate the levels of serum liver enzymes such as alanine aminotransferase (EC 2.6.1.2), aspartate aminotransferase (EC 2.6.1.1), and alkaline phosphatase (EC 3.1.3.1) [41,42,43]. In animal studies using mouse models, IH exposure caused hepatic steatosis, necrosis of hepatocytes, and inflammation of the liver with neutrophil accumulation and collagen deposit. The mechanisms involve increases in proinflammatory cytokines (such as interleukin [IL]-1β, IL-6, tumor necrosis factor-α [TNF-α], and chemokine [C-X-C motif] ligand 2 [CXCL2]) and oxidative stress, resulting in DNA damages and the apoptosis of hepatocytes [41,42,43]. de Rosa et al. showed that after 3–5 weeks of IH in C57BL/6 mice, both hypoxia-inducible factor (HIF)-1 and phosphorylated (activated) nuclear factor-κB (NF-κB) were upregulated in the liver [44]. IH was also shown to increase proinflammatory cytokines, such as TNF-α and CXCL2, in obese mice exposed to four weeks of IH. Another study observed increases in NF-κB activation and liver proinflammatory cytokines such as IL-1β, IL-6, and CXCL2 in lean mice exposed to longer periods of IH [41]. IH also results in upregulation of nitric oxide synthase 2 (NOS2: EC 1.14.13.39) and reduced activity of liver antioxidant enzymes such as superoxide dismutase (EC 1.15.1.1) and catalase (EC 1.11.1.6), both of which can contribute to inducing DNA damage and apoptosis [44]. Although IH induced several inflammatory responses in the liver, which particular cells (whether hepatocytes, hepatic stellate cells, sinusoid endothelial cells, Kupffer cells, pit cells, or intrahepatic biliary epithelial cells) are primary targets in IH-induced liver damage remains elusive. Liver damage by IH is thought to be significantly involved in the pathogenesis of non-alcoholic fatty liver disease (NAFLD). NAFLD is strongly associated with obesity, diabetes, and metabolic syndrome (obesity, hyperlipidemia, type 2 DM, and high blood pressure) [42]. With the global trend toward obesity, the incidence of NAFLD in obesity has risen rapidly to almost 70% and is recognized as the hepatic component of metabolic syndrome [45]. A recent prospective study showed that NAFLD occurs in more than 70% of patients with type 2 DM [46] and can be regarded as a risk factor for type 2 DM, independent of age or other factors such as obesity [47]. NAFLD patients usually have hepatic insulin resistance, which is associated with NAFLD-related lipid accumulation, inflammation, endoplasmic reticulum stress, and oxidative stress [48]. Moreover, hepatic insulin resistance is the key cause of impaired fasting glucose, which contributes substantially to the development of type 2 DM [49]. IH exposure in mice increases hepatic lipogenic enzymes such as stearoyl-coenzyme A desaturase-1 (EC 1.14.19.1) via the upregulation of sterol regulatory element–binding protein-1 and high-density lipoprotein receptor [50], leading to the development of NAFLD and metabolic syndrome. In another study, IH was reported to be associated with fibrosis and inflammation of the liver but not with macrophage accumulation [51]. Animal studies, especially in obese mice model, strongly support the pathophysiological contribution of IH to the progression of NAFLD [42,52].

### 3.2. Effects of IH on Hepatic Glucose Metabolism

**Figure 2:**
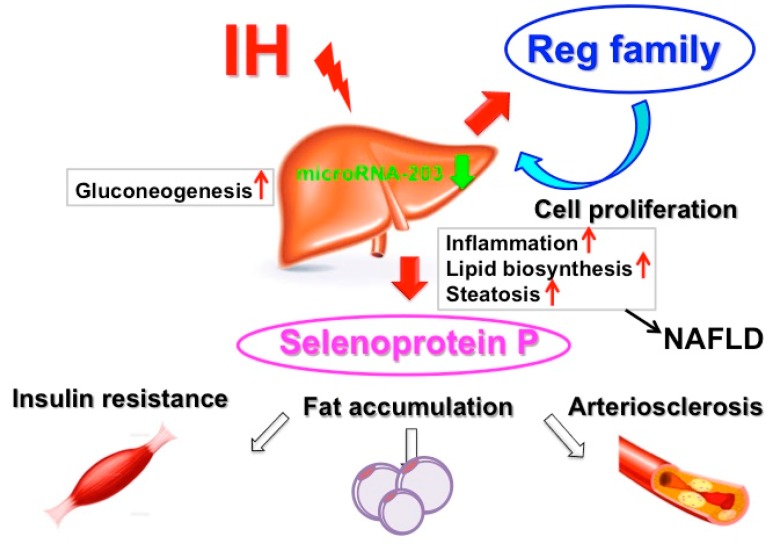
A possible mechanism for IH-induced diabetes mellitus (DM) and its complications. IH upregulates hepatokines such as selenoprotein P to increase insulin resistance and HIP/PAP [59] to proliferate such hepatocytes via downregulation of microRNA-203, resulting in insulin resistance, fat accumulation, and arteriosclerosis.

The direct mechanism by which IH affects hepatic glucose metabolism is not well understood. Savransky et al. report that IH upregulates glucose production, as supported by observation of higher glycogen content [41,42]. Furthermore, IH increases not only glucose supply from hepatocytes but also gene expression of several gluconeogenic enzymes such as phosphoenolpyruvate carboxykinase (EC 4.1.32) and glucose 6-phosphatase (EC 3.1.3.9) in the liver, contributing to fasting hyperglycemia and development of type 2 DM [53]. Gu et al. report that IH disrupts glucose homeostasis in hepatocytes in an insulin-dependent and independent manner [54]. Recently, several proteins that are exclusively or predominantly secreted by the liver, called hepatokines, were confirmed as directly affecting glucose and lipid metabolism [55,56]. For example, selenoprotein P, a type of hepatokines, is correlated positively with insulin resistance and could be a therapeutic target for type 2 DM [57]. In cellular studies using human and rat hepatocytes, IH exposure causes upregulation of mRNAs in selenoprotein P but not in α2 HS-glycoprotein, angiopoietin-related growth factor 6, fibroblast growth factor 21, leukocyte cell-derived chemotaxin 2, Lipasin, and sex hormone-binding globulin, or hepatocarcinoma-intestine-pancreas/pancreatitis-associated protein (HIP/PAP), but in the other Reg family (RΕG Iα, REG Iβ, REG III, and REG IV) it happened in hepatocytes via downregulation of microRNA-203. HIP/PAP, a Reg family member [39], was reported to be a hepatocyte mitogen [39,58,59] and the small interfering RNA for HIP/PAP attenuated the IH-induced hepatocyte proliferation [59]. It may be that IH stress upregulates the levels of selenoprotein P in human hepatocytes to accelerate insulin resistance and the levels of HIP/PAP mRNAs to proliferate such hepatocytes via the microRNA-203 mediated mechanism [59] (Figure 2).

## 4. Intermittent Hypoxia and Adipose Tissue

### 4.1. Insulin Resistance Induced by Lipolysis of the White Adipose Tissue (WAT)

Obesity is strongly associated with SAS and exerts many of its complications in cardiovascular and metabolic systems through the action of WAT. Recently, physiological and pathophysiological roles of WAT have been explored [60,61,62]. WAT plays a major role in insulin resistance through the release of free fatty acids (FFAs) during lipolysis [63], and these induce insulin resistance via their effects not only on adipose tissue but also on liver and muscle cells. As adipose tissue accumulates fat, it increases in volume and consequently become much more hypoxic [63,64,65]. In response to this hypoxia, the WAT undergoes apoptosis and necrosis in cell death. Adipocyte death may contribute to elevation of FFAs in circulation because dead adipocytes may release FFAs into bloodstream. Various studies have shown that in obese humans or animals WAT is more hypoxic than in lean controls [66,67]. Hypoxia is a key component in modulating the inflammatory responses of WAT [63,64]. When adipocyte hypertrophy occurs and the adipocyte size exceeds the oxygen diffusion capacity, the adipose tissue regional perfusion is reduced, and the adipocytes are in hypoxic condition [63].

### 4.2. Inflammation in Adipose Tissue by IH

Hypoxic stress accelerates adipocyte inflammatory signal transduction pathways in which HIF-1α and NF-κB play important roles [65]. HIF-1 is recognized as one of master transcription factors for numerous genes affecting various cellular and developmental processes, including angiogenesis, cellular and systemic metabolism, vascular tone, and cell survival [68]. It has recently been shown that HIF-1 activation occurs at the onset of obesity as a response to relative tissue hypoxia, leading to a state of insulin resistance in adipocytes, with adipose tissue inflammation and metabolic dysfunction [69]. Hypoxia has also been shown to inhibit insulin signaling by decreasing insulin receptor tyrosine-phosphorylation and glucose transport in response to insulin, affecting adipocytes in a HIF-1 dependent manner [70]. Adipose HIF-1 activation, HIF-1-mediated angiopietin-like 4 expression, and inhibition of lipoprotein lipase (EC 3.1.1.34) by IH have also been demonstrated in apolipoprotein E-deficient lean mice [14]. Hypoxia increases macrophage infiltration in adipose tissue and the expression/secretion of IL-6, IL-8, and TNF-α from adipose tissue [24]. A possible explanation is proposed that primary human adipocytes exposed to IH in vitro are significantly more sensitive to the stimulus than other primary cells through the activation of downstream NF-κB pathway by which the expression of multiple inflammatory mediators, such as TNF-α and C-C motif chemokine ligand 2 (CCL2), play important roles in insulin resistance [71].

**Figure 3:**
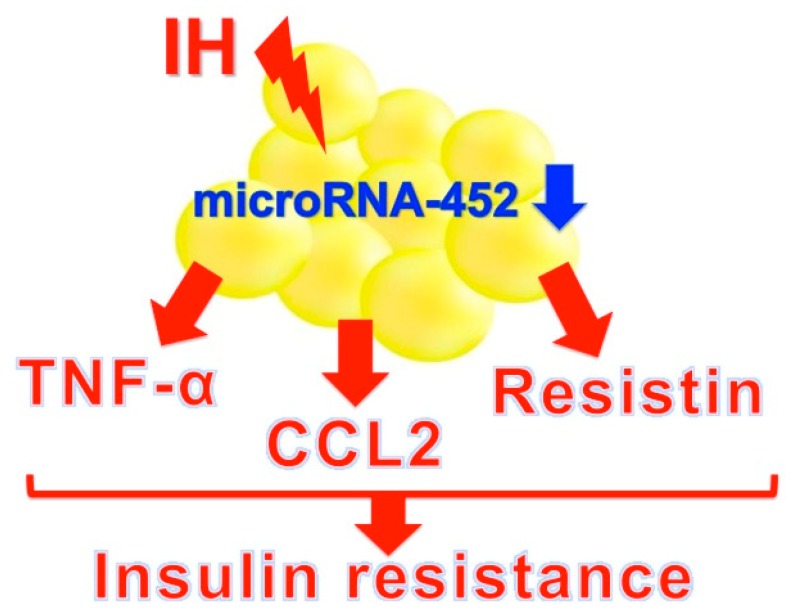
A possible mechanism of IH-induced insulin resistance via adipokines. IH stress upregulates adipokines such as tumor necrosis factor-α (TNF-α), C-C motif chemokine ligand 2 (CCL2), and resistin via downregulation of microRNA-452 in adipocytes to increase insulin resistance [75].

Pathological changes in adipose tissues are characterized by infiltration of macrophages and other immune cells, including T-lymphocytes and mast cells. Macrophages are polarized toward the pro-inflammatory subtype M1 and are arranged in a crown-like structure around necrotic adipocytes. M1 macrophages produce various pro-inflammatory cytokines, such as IL-6 and TNF-α and express NOS2 [72,73]. The resulting adipose tissue inflammation leads to a release of FFAs which activate various signaling pathways, including c-Jun N-terminal protein kinases, the inhibitor of NF-κB kinase subunit β and protein kinase R, collectively resulting in impairment of the insulin-signaling pathway, with the downstream consequence of insulin resistance and metabolic dysfunction [74]. Recently, Uchiyama et al. reported that IH upregulated mRNAs and the proteins TNF-α, CCL2, and resistin in human and rodent adipocytes via downregulation of microRNA-452 [75]. TNF-α plays a key role in obesity-related insulin resistance and increased TNF-α levels contribute to impaired glucose homeostasis [76]. CCL2, also referred as monocyte chemoattractant protein-1, is a key regulator of monocyte infiltration in adipose tissue and plays a central role in the development and maintenance of chronic adipose tissue inflammation and insulin resistance [76,77]. Resistin is a pro-inflammatory adipokine and was initially named because of its relationship to insulin resistance in rodents [72,77]. Such inflammatory adipokines play an important role in the onset and progression of insulin resistance and type 2 DM (Figure 3). Additionally, as TNF-α, CCL2, and resistin were reported to associated with inflammation and macrophage infiltration [78,79,80], adipocyte dysfunction in vivo can be achieved by a combination of TNF-α, CCL2, and resistin from IH-exposed adipocytes and other inflammatory mediators such as IL-6 from infiltrated macrophages or lymphocytes.

## 5. Intermittent Hypoxia and Skeletal Muscles

Skeletal muscles are responsible for the majority of insulin-sensitive glucose uptake via glucose transporter 4 (solute carrier family 2, facilitated glucose transporter member 4). In vivo research presents data that are conflicting, some suggesting that IH induces insulin resistance while others show improvements in insulin sensitivity. A study using a rodent model of IH showed not only decreases in whole-body insulin sensitivity but also reduced glucose utilization and insulin sensitivity in the *musculus soleus*, suggesting a clear reduction in glucose metabolism with reduced uptake in the muscle. The impact of IH was most pronounced in oxidative muscle fibers (*musculus soleus*), while glycolytic muscle (*musculus vastus lateralis*) and mixed oxidative and glycolytic (*musculus gastrocnemius*) fibers were relatively unaffected. Thus, glucose uptake in oxidative muscle tissue is significantly impaired by IH and this effect appears independent of obesity [22]. On the other hand, Mackenzie et al. [81] showed that acute hypoxic exposure increased insulin sensitivity in individuals with type 2 DM, and ten nights of moderate hypoxic exposure improved insulin sensitivity in obese males [82]. The question whether IH causes insulin resistance or not is a complex one. There are few studies that have examined the effect of IH on glucose uptake and metabolism in skeletal muscle. Recently, it was found that muscle cells express and secrete several cytokines, and these are called myokines [83]. Most recently, IH was shown to upregulate some myokines, such as IL-8, osteonectin (also known as secreted protein acidic and rich in cysteine), and myonectin (also known as C1q/TNF-related protein 15 or erythroferrone), which are all involved in inflammation and glucose metabolism, via transcriptional activation of the myokine genes in human and mouse muscle cells [84]. IH-induced upregulation of myokines could be an important research target for an understanding of why and how IH induced glucose intolerance develops.

## 6. Intermittent Hypoxia and Nervous System

Sympathetic excitation induced by IH has been extensively studied and is widely recognized to contribute to the IH-induced cardiovascular complications [85]. In addition, catecholamines, secreted from hypothalamic-pituitary-adrenal (HPA) system, are known to reduce insulin sensitivity and insulin-mediated glucose uptake in peripheral organs and tissues [86,87]. Furthermore, elevated sympathetic excitation seems to be sustained during the day as well as being evident during sleep, even if day time breathing seems to be normal in patients with SAS [88,89]. Several studies have clearly demonstrated the elevation in sympathetic activity and its associated increase in blood pressure in SAS patients was improved following short-term continuous positive airway pressure (CPAP) therapy [90,91,92,93]. As catecholamines are widely known to reduce peripheral insulin-sensitive glucose uptake and to increase insulin resistance [86], these findings strongly suggest that IH causes insulin resistance in peripheral organs/tissues via the increased release of catecholamines from sympathetic neural system. Although SAS and obesity are strongly related, it remains unclear which is the trigger. Shobatake et al. show that IH stress upregulates the mRNA levels of major appetite regulatory peptides, proopiomelanocortin (POMC) and cocaine- and amphetamine-regulated transcript (CART), in human neuronal cells. IH can have an anorexigenic effect on SAS patients through the transcriptional activation of POMC and CART in the central nervous system via the activation of GATA transcription factors [94]. Recently, IH was shown to upregulate anorexigenic hormone genes (peptide YY [PYY], glucagon-like peptide-1 [GLP-1], and neurotensin) in human and rodent enteroendocrine cells via epigenetic modification of chromatin structure [95]. Therefore, IH shows anorexigenic effects not only through the central nervous system via expression of POMC and CART but also through the peripheral nervous system via upregulation of PYY, GLP-1, and neurotensin.

## 7. Therapeutic Interventions for IH/SAS and Type 2 DM

Although there are various treatments for SAS patients, CPAP therapy remains the gold standard for treating SAS. Randomized, placebo-controlled trials have indicated that CPAP shows significant improvement in quality of life of SAS patients. Several alternatives to CPAP therapy may be considered, including mandibular advancement devices for increasing airway diameter through soft tissue displacement, surgeries to the upper airway, maxillomandibular advancement, and gastric bypass surgery (mainly Roux-en-Y gastric bypass) for weight loss in appropriately selected patients. However, the impact of CPAP therapy on DM is less clear in research measuring the variable markers of insulin sensitivity and insulin resistance. Several randomized controlled trials have reported distinct improvements in metabolic control (insulin sensitivity/glucose tolerance) in patients with SAS who were treated with CPAP as compared to sham-CPAP treated patients [96,97,98]. Moreover, many studies have shown improvements in glycemic control such as hemoglobin A1c (HbA1c) after three months of CPAP therapy [99,100,101]. Nevertheless, in a recent meta-analysis of randomized controlled studies that examined the effects of CPAP on glycemic control measurements, CPAP does not show decreases in HbA1c level or body mass indices in patients with SAS and type 2 DM, but it does show improved insulin sensitivity [102]. Hecht et al. reported that CPAP neither influenced plasma insulin levels nor the homeostasis model assessment index, adiponectin levels, or HbA1c value as part of their meta-analysis of variables in SAS and they found that CPAP did not improve insulin sensitivity [103]. Lindberg *et al*. [104] reported improvement in serum fasting insulin levels but not in HbA1c levels with CPAP therapy. These controversial results described here may be caused by differences in assessment methods for insulin sensitivity, variations in study population characteristics such as age, sex, race, severity of SAS, incorrect attachment of CPAP device, and duration of CPAP therapy. However, these findings indicate that CPAP therapy for SAS patients can lead to significant improvement in glucose tolerance and control, even in type 2 DM and pre-DM. As the molecular bases of SAS/IH are described, the efficacies of CPAP and other therapies for SAS/IH-induced insulin resistance, for DM, and for SAS itself, should also be assessed in relation to molecular markers.

## 8. Conclusions

**Figure 4:**
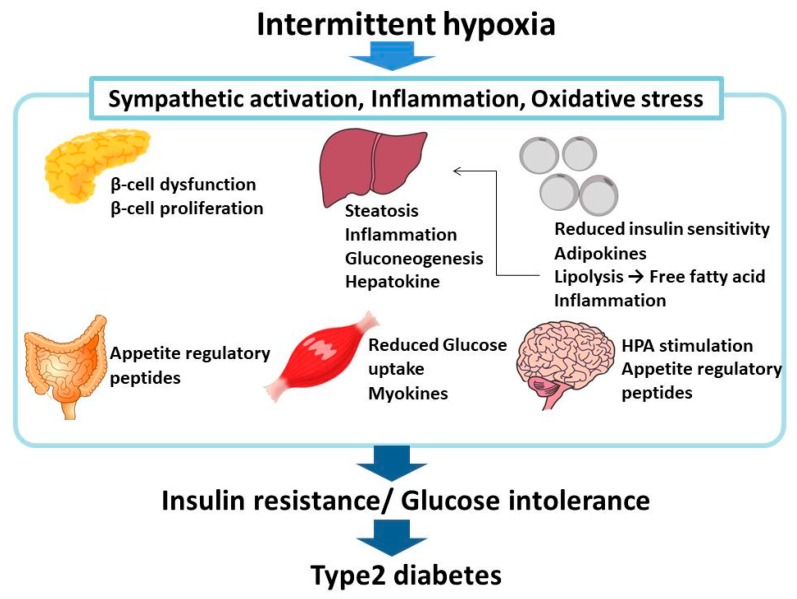
Relationship between SAS/IH and insulin resistance/glucose intolerance/type 2 DM. IH in SAS patients induces a number of cellular responses, most of which worsen insulin sensitivity and glucose tolerance (e.g., reduction of glucose-induced insulin secretion [31], upregulation of selenoprotein P [59], upregulation of adipokines such as CCL2, TNF-α, and resistin [75], and upregulation of myokines such as IL-8, osteonectin, and myonectin [84]), leading to type 2 DM.

Recently, there has been great medical and scientific interest in the interaction between SAS/IH and metabolic dysfunction. SAS is commonly found in patients with type 2 DM. Recent research indicates that SAS could contribute to impaired glucose metabolism through the combined effects of sleep fragmentation, sympathetic excitation, and oxygen stress induced by IH. IH plays a key role not only in the pathogenesis of SAS but also in the pathophysiology of SAS-induced metabolic disorders such as insulin resistance and type 2 DM. It is therefore important for the experimental research to use IH models in animals and in vitro. IH plays a pivotal role in the development of glucose metabolic dysfunction in SAS and contributes through multiple pathways to the complications of SAS, including obesity (Figure 4). In order to clarify the mechanisms underlying these processes, molecular, clinical, and translational research in vitro and in vivo is urgently required.

## Abbreviations

Bax	Bcl-2-associated X protein
Bcl-2	B cell lymphoma 2
CART	Cocaine- and amphetamine-regulated transcript
cADPR	Cyclic ADP-ribose
CPAP	Continuous positive airway pressure
CCL2	C-C motif chemokine ligand 2
CXCL2	Chemokine (C-X-C motif) ligand 2
DM	*Diabetes mellitus*
FFAs	Free fatty acids
GLP-1	Glucagon-like peptide-1
HbA1c	Hemoglobin A1c
HIF	Hypoxia-inducible factor
HIP/PAP	Hepatocarcinoma-intestine-pancreas/pancreatitis-associated protein
HPA	Hypothalamic-pituitary-adrenal
IH	Intermittent hypoxia
IL	Interleukin
NAFLD	Non-alcoholic fatty liver disease
NF-κB	Nuclear factor-κB
NOS2	Nitric oxide synthase 2
POMC	Proopiomelanocortin
PYY	Peptide YY
Reg	Regenerating gene
SAS	Sleep apnea syndrome
SNP	Single nucleotide polymorphism
TNF-α	Tumor necrosis factor-α
WAT	White adipose tissue
